# Virus-Induced Asthma/Wheeze in Preschool Children: Longitudinal Assessment of Airflow Limitation Using Impulse Oscillometry

**DOI:** 10.3390/jcm8091475

**Published:** 2019-09-16

**Authors:** George N Konstantinou, Nikolaos G Papadopoulos, Emmanouel Manousakis, Paraskevi Xepapadaki

**Affiliations:** 1 424 General Military Training Hospital, 56429 Thessaloniki, Greece; 2 Allergy Department, 2nd Pediatric Clinic, National and Kapodistrian University of Athens, 11527 Athens, Greece; 3 Division of Infection, Immunity & Respiratory Medicine, University of Manchester, Manchester M13 9PL, UK

**Keywords:** lung function, bronchodilation, resistance, obstruction, reproducible, inflammation, spirometry

## Abstract

Several researchers have assessed the utility of Impulse Oscillometry System (IOS) in diagnosing and evaluating the severity of respiratory diseases in childhood, but none has investigated the impact of the fluctuations of IOS parameters in an individualized manner. In this two-year prospective study, we aimed to longitudinally evaluate changes in airflow limitation and bronchodilator responsiveness in steroid-naïve four- to six-year-old children during a virus-induced wheezing episode, with IOS pulmonary resistance parameters set at 5 (R5) and 20 (R20) Hz. Moreover, feasibility and reproducibility, in addition to the diagnostic properties of these parameters were examined. Lung function was assessed every six weeks (baseline), within the first 48 h following an acute wheezing episode (Day 0), after 10, and after 30 days. Forty-three out of 93 recruited children (4.5 ± 0.4 years old) experienced a wheezing episode during the study period. All children were able to perform the IOS effort in an acceptable and highly reproducible manner. R5 and R20 fluctuated independently of atopy, age, height, and weight. On Day 0, R5 values were significantly lower than the respective baseline values and returned to individual baseline levels within 10 days. Post-bronchodilation R5 values were similar to the baseline ones, reflecting a reversible airway obstruction on Day 0. Response to bronchodilation (ΔR5) was significantly more pronounced on Day 0. ΔR5 values lower than −20.5% had a sensitivity of 70% and a specificity of 76% and could accurately identify up to 75% of the examined preschoolers. This study provides evidence in favor of the objective utility of IOS as an easy, highly reproducible, and sensitive technique to assess clinically significant fluctuations and bronchodilation responses suggestive of airflow limitation. Reference values although necessary are suboptimal, utilizing the personal best values as personal reference is useful and reliable.

## 1. Introduction

Asthma is the most common chronic lower respiratory disease in childhood throughout the world. Current guidelines are highly in favor of documenting reversible airflow obstruction as a cardinal characteristic of asthma, both for the diagnosis and the subsequent monitoring of asthma, preferably prior to controller treatment, in all age groups [1]. The most widely used pulmonary function test is spirometry, which estimates lung volumes by rapid and maximal inspiratory and expiratory maneuvers that are often difficult to perform even in older children.

In contrast, the Forced Oscillation Technique (FOT) superimposes small air pressure perturbations on the natural breathing of a subject to measure the mechanical properties of the lungs [2]. The Impulse Oscillometry System (IOS), based on the aforementioned technique, measures the resistance and reactance of the respiratory system, thus providing an indirect analysis of lung function through the use of short pulses (impulses) of acoustic waves—most commonly over a range of frequencies (5Hz to 20Hz)—applied at the mouth, during spontaneous, quiet breathing [3]. IOS has been used to distinctively quantify the airflow limitation in the central and peripheral airways [4,5]. Low oscillation frequencies like 5 Hz can penetrate the periphery of the bronchial tree (diameter <2 mm) and, therefore, resistance of the respiratory system at 5 Hz (R5) reflects obstruction in both the peripheral and the central airways. On the contrary, higher frequencies cannot be transmitted distally. Thus, resistance of the respiratory system at 20 Hz (R20) reflects the proximal airways resistance. The change in resistance from low to high-frequency ranges (e.g., R5 minus R20, R(5−20)) has been identified as an index of the peripheral airways resistance only, and has been used as a potential marker of small airways obstruction [6].

IOS has been developed as a patient-friendly lung function test that minimizes demands on the patient and requires only passive cooperation with normal breathing through the mouth. It has been successfully used for assessing lung function and asthma control in healthy and asthmatic children [5,6], including preschoolers, and in patients who recently underwent surgery or are unable to perform spirometry, both as an adjunct or even alternative to standard spirometry [4,7,8].

Pediatric reference values and positive bronchodilation responses indicating peripheral air trapping have been standardized and published [9]. R5 is the main parameter to assess bronchodilation. However, no consensus has been reached on the optimal cutoff values that could discriminate patients from healthy individuals. These values vary between 20 and 50% and have been used to diagnose and evaluate the severity of chronic respiratory diseases in childhood or compare to a number of techniques routinely used to assess lung function [10,11,12,13,14]. Nevertheless, none of the studies has prospectively followed either baseline fluctuations of IOS parameters or fluctuations in the course of virus-induced wheeze episodes in preschool children.

In this prospective study, we aimed to longitudinally evaluate changes in airflow limitation and bronchodilator responsiveness by means of the main IOS resistance parameters, in steroid-naïve four- to six-year-old children during the course of a virus-induced wheezing episode. Moreover, IOS feasibility and reproducibility, in addition to the diagnostic properties of the main IOS resistance parameters were examined.

## 2. Methods

### 2.1. Study Population

Children four to six years of age from the outpatient clinics of the Allergy Department in the Second Pediatric Clinic of the National and Kapodistrian University of Athens with a previous diagnosis of “episodic viral wheeze” according to the European Respiratory Society Task Force [15] or “virus-induced asthma” according to the Practical Allergy (PRACTALL) Consensus Report [16] were invited to participate. Patients were eligible if they (1) had been given this diagnosis within the 12 months preceding the index visit, (2) had at least one mild wheezing episode (based on reference [16]) in the 12 months preceding the index visit, and (3) if—according to their medical records—there were prescribed inhaled β-agonists, inhaled corticosteroids or montelukast, but did not require hospitalization. Patients receiving inhaled corticosteroids or montelukast on a regular or episodic basis were recruited after a minimum 14-week wash-out period. For the present study, children were required to be able to perform acceptable and repeatable IOS maneuvers after proper training. Since the protocol per se included both spirometric and IOS pulmonary function testing, children were required to be able to perform a technically acceptable maximal expiratory flow/volume effort with a forced expiratory time (FET) of 0.5 s or greater [17]. The study was approved by the institutional ethics committee, and written informed consent was obtained from all parents. The outcomes of additional methods and the baseline characteristics of the study population recruited have been previously described [18].

### 2.2. Study Design

Upper and lower airway symptoms and medication use were recorded daily by the parents on diary cards. Parents were advised to contact the study physicians to arrange an appointment within 48 h after the child started wheezing or coughing, had difficulty breathing or nighttime awakening due to breathing difficulties. The children were followed up at regular six-week intervals, until either they had a wheezing episode or reached their sixth birthday. Study physicians were responsible to evaluate the diary cards and to perform all IOS maneuvers at baseline, during the first 48 h following a physician-diagnosed wheezing exacerbation (day 0), and then 10 (day 10) and 30 (day 30) days following the initiation of the episode. During the episodes, children were added as a study observation if they could be controlled with 200–400 μg of salbutamol, 3–4 times a day and as needed.

### 2.3. Allergic Sensitization

Levels of serum-specific IgE (ImmunoCAP; Phadia AΒ, Uppsala, Sweden) to a panel of locally relevant common aeroallergens (*Dermatophagoides pteronyssinus*, *Dermatophagoides farinae*, cat dander, dog epithelium, grass pollen, *Cladosporium* species, *Aspergillus* species, *Alternaria* species, olive, cypress, and wall pellitory pollen), and food allergens (hen’s egg, cow’s milk, nuts, and peanut) were measured either after the wheezing episode or at the age of six years for those children without a wheezing episode during the study. A patient was classified as atopic if at least one allergen-specific IgE was greater than 0.7 IU/mL.

### 2.4. Lung Function Test Maneuvers

IOS was performed using MasterScreen IOS (Jaeger, Würzburg, Germany), applying a standardized protocol based on manufacturer’s instructions. The system was calibrated through several full strokes of a single volume (3 L) of air at different flow rates, which were verified with a reference resistance device (2.0 cm H_2_O/L/sec) supplied by the manufacturer. Children withheld the use of short-acting bronchodilators at least 6–8 h prior to testing, which was performed and analyzed in accordance with European Respiratory Society (ERS)/American Thoracic Society (ATS) guidelines [17,19]. IOS testing was performed before the spirometry in order to prevent potential bronchoconstriction [7]. Prior to testing, the child was familiarized with the procedure and was placed in a relaxed standing position, with the head in a neutral or slightly extended position. Subsequently, the patient was instructed to breathe normally during the test, making a tight seal with their lips around the mouthpiece. A nasal clip was also used, while the cheeks were firmly supported. After a short sampling period to ensure compliance, three to five efforts lasting 20–60 s were recorded. The 20–60 s period provides the min-max limits of an effort to record an artifact-free maneuver. In case there was no evidence of coughing, swallowing, vocalization or breath-holding causing artifacts during this period, the trial was saved. Pulmonary resistance (R) at frequencies of 5 Hz (R5) and 20 Hz (R20) were calculated with the pre-installed software and assessed by researchers. Each visit-observation consisted of an optimum of three reproducible maneuvers. Reproducibility was defined as R5 within 10% of highest obtained value. R5 and R20 measurements from the three saved reproducible efforts for each IOS parameter were averaged. Calculations of the R(5−20) were assessed with an algorithm based on the equation R5 − R20.

Spirometry was performed with the children in the standing position by using a nose clip and an incentive animation. Results were reported only if at least two technically acceptable curves (<8 maneuvers per visit), as determined according to standard criteria [17], with a FET ≥0.5 s, were obtained.

In order to assess airway reversibility, a short-acting bronchodilator was administered (four puffs of albuterol, 100 μg each) using a spacer. After 15 min, IOS was repeated. Predicted values for R5 and R20 were based on gender and height according to the equipment’s default normal reference values, as recommended by the manufacturer, based on existing reference values [20,21].

### 2.5. Statistical Analysis

Since all the analyzed parameters were normally distributed, descriptive statistics for continuous variables are presented as means ± standard deviation (SD). Student’s t-tests were used to compare binary outcomes at the same time point. Student’s paired t-tests were used for between-time point comparisons of the same variable. Associations between categorical data were assessed using the Pearson χ^2^ test. Analyses across these time points were performed with generalized estimating equations (GEE) after adjusting for known confounders, such as height, age, and atopy. For the period from day 0 to day 30, all variables were treated as time-dependent, except age, sex, atopy, and height.

A logistic regression analysis was performed among the binary outcome “having” or “not having” a symptomatic wheezing episode, the resistance parameter of interest, and the above-mentioned potential confounders. The same analysis was performed either by using the data from the cohort of patients with a wheezing episode alone or after pooling data from both the children with and without a wheezing episode. Between and within individuals, variability was taken into account in the analysis of the pooled data. Predictor levels corresponding to “having a wheezing episode” with a probability of ≥95% (95% predictive decision points) were identified, and so were their performance characteristics (sensitivity, specificity, positive and negative predictive values calculated). Receiver operating characteristic (ROC) analyses and curves were fitted to identify “optimal decision points,” and the area under the curve (AUC) was calculated to compare the accuracy of each analyzed resistance parameter. Additional information can be found in this article’s supplementary data.

## 3. Results

All 98 consecutively examined children were able to perform an acceptable IOS effort and consent to be included in the study. Five of them were unable to achieve an acceptable spirometric maneuver with FET ≥0.5 s, even after three visits of continuous training efforts, and were excluded. All 93 finally recruited children (mean age 4.5 ± 0.4 years) were able to perform an IOS effort both at baseline and during the wheezing episodes. None of the children needed more than three demonstrations and an equal number of efforts during their initial visit to accomplish a technically correct IOS maneuver. Among them, 49 (52.7%) were able to perform a correct spirometric maneuver during the first visit, 32 (34.4%) needed two visits, and the remaining 12 (12.9%) needed three visits to be trained well enough to perform an acceptable and repeatable spirometric maneuver. Two out of these 12 were not able to perform spirometry and an acceptable IOS effort during their first wheezing episode.

Regular follow-up visits occurred every 40 ± 4 days. Four children were lost to follow-up. Among the remaining 89 children, 43 had at least one wheezing episode 0.6 ± 0.3 years after recruitment. This corresponds to a median of five visits for each patient. According to the study protocol, among these regular visits, the very last one was considered the baseline.

Following initial assessment at the beginning of an episode (day 0), the children were re-evaluated 10 ± 1 days (day 10) and 30 ± 3 days (day 30) later. None of the children reported wheezing unrelated to an apparent respiratory tract infection.

There was no significant seasonality for the wheezing episodes except the period from early May until late September (fewer episodes).

The variability of R5 and R20 among different visits was also estimated. Measurements at regular visits and during the course of a wheezing episode were examined. Pre- and post-bronchodilation R5 variability ranged from 2.9% to 33.7% (median: 15%, upper 95% percentile: 32.3%) and 1% to 27.7% (median 10.2%, upper 95% percentile 23.4%), respectively. Pre- and post-bronchodilation R20 variability ranged from 3% to 19.2% (median 8%, upper 95% percentile: 18.5%) and 1.7% to 25% (median 7%, upper 95% percentile: 16.5%).

The baseline demographic and somatometric characteristics and IOS measurements were similar between atopic children, non-atopic children, and children completing the study without any wheezing episode. Bronchodilation responses assessed by R5 and R20 did not differ either (Table 1 and Supplementary Table S1).

**Figure 1:**
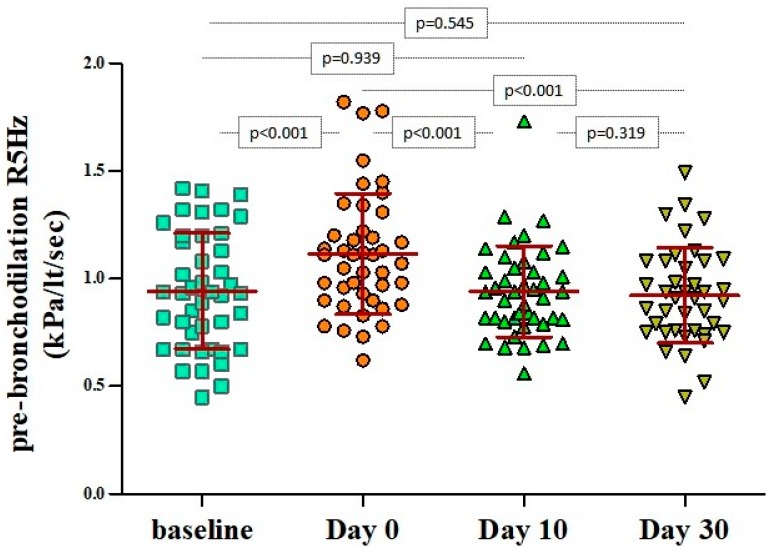
Pairwise comparisons of R5Hz values at baseline, on day 0 (beginning of the wheezing episode), and 10 and 30 days after. The bars and in-between lines represent the mean and standard deviation (SD). All pairwise comparisons are presented with *p*-values that have been estimated with paired Student’s *t*-test.; Pulmonary resistance (R) at 5 Hz (R5).

During the first 48 h from the beginning of a wheezing episode (day 0), pre-bronchodilation R5 values were significantly higher than their respective baseline values (1.114 ± 0.280 kPa/lt/sec vs 0.943 ± 0.269 kPa/lt/sec, *p* < 0.001). The aforementioned measurements returned to baseline levels within 10 days from the initiation of the episode (Figure 1). There were no significant differences in respect to the atopic status at all time points (Supplementary Table S2).

A similar fluctuation pattern, independent of the atopic status, was recorded for R(5−20) values, namely day 0 vs baseline: 0.314 ± 0.163 kPa/lt/sec vs 0.186 ± 0.115 kPa/lt/sec, respectively, *p <* 0.001. In respect to the R20, a small but significant increase was noted on day 0 compared to baseline, namely 0.801 ± 0.162 kPa/lt/sec vs 0.757 ± 0.191 kPa/lt/sec, respectively, *p* = 0.048.

R5 and R20 were not found to be significantly related to age, gender, and somatometric measures (cross-sectional logistic regression models at each time point and longitudinal GEE models). Gender and height, however, are used by the predicted equations to estimate the equipment’s default normal reference values. Pairwise correlation coefficients, although statistically significant, did not indicate strong correlations between reference/predicted values and actual values for both pre-bronchodilation R5 and R20 with Pearson’s correlation coefficients 0.522 vs 0.415 respectively, *p* < 0.001 (Supplementary Figures S1 and S2). The same applied for the post-bronchodilation values with Pearson’s correlation coefficients 0.496 and 0.308, respectively, *p* < 0.001 (Supplementary Figures S3 and S4). Independence of age, height and weight and suboptimal reference/predicted values suggested that, at least for the examined ages (four to six years of age), the personal best baseline measurement (lowest values) should be the reference values for each individual. Therefore, the reference/predicted values were not taken into consideration in any of the performed calculations. Additional information can be found in this article’s supplementary data.

All post-bronchodilation values were significantly different in relation to the respective pre-bronchodilation at all time points for both R5 and R20 (Table 2). Post-bronchodilation R5 values on day 0 were similar to the baseline pre-bronchodilation values, reflecting the reversible airway obstruction occurring at the beginning of a wheezing episode both in atopic and non-atopic children (Table 2).

**Figure 2:**
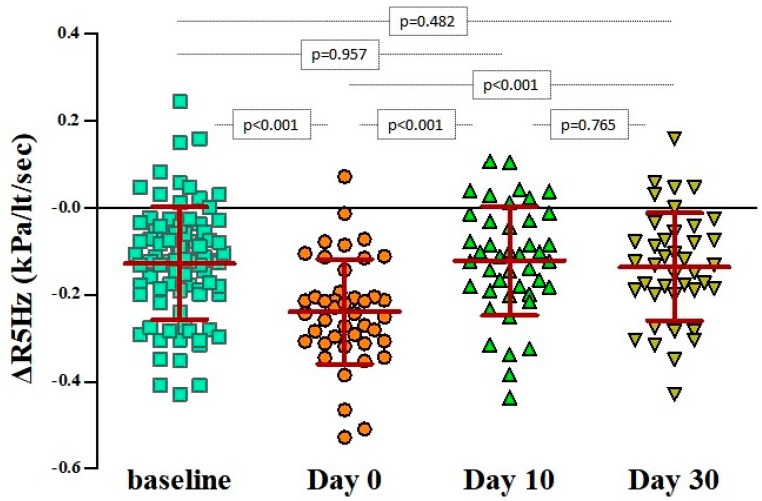
Pairwise comparisons of ΔR5Hz values at baseline, on day 0 (beginning of the wheezing episode), and 10 and 30 days after. The bars and in-between lines represent the mean and SD. All pairwise comparisons are presented with *p*-values that have been estimated with paired Student’s t-test.; Pulmonary resistance (R) at 5 Hz (R5); ΔR5Hz = (R5Hz _post-bronchodilation_ – R5Hz _pre-__bronchodilation_) / R5Hz _pre-bronchodilation_

**Figure 3:**
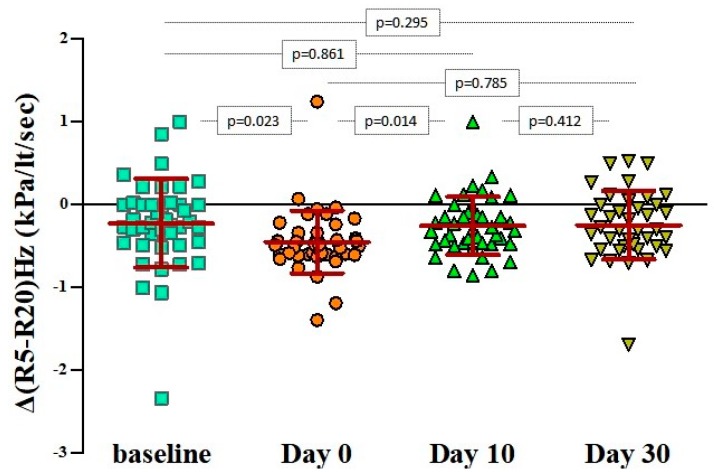
Pairwise comparisons of Δ(R5Hz−R20Hz) values at baseline, on day 0 (beginning of the wheezing episode), and 10 and 30 days after. The bars and in-between lines represent the mean and SD. All pairwise comparisons are presented with *p*-values that have been estimated with paired Student’s t-test. Pulmonary resistance (R) at 5 Hz (R5) and 20 Hz (R20); ΔR(5−20)Hz = ((R5Hz−R20Hz)_post-bronchodilation_ − (R5Hz−R20Hz)_pre-bronchodilation_) / (R5Hz−R20Hz)_pre-bronchodilation._

**Figure 4:**
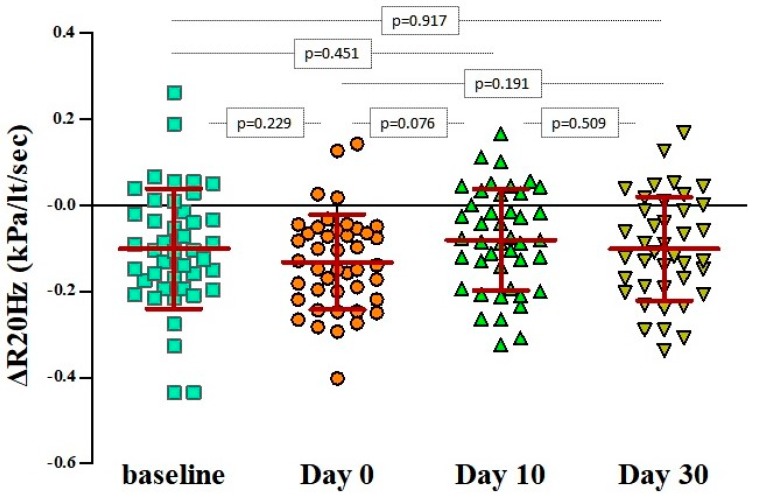
Pairwise comparisons of ΔR20Hz values at baseline, on day 0 (beginning of the wheezing episode), and 10 and 30 days after. The bars and in-between lines represent the mean and SD. All pairwise comparisons are presented with *p*-values that have been estimated with paired Student’s t-test. Pulmonary resistance (R) at 20 Hz (R20); ΔR20Hz = (R20Hz_post-bronchodilation_ – R20Hz_pre-bronchodilation_) / R20Hz_pre-bronchodilation._

In particular, bronchodilation responses assessed by ΔR5 were significantly more pronounced only during the first 48 h from the initiation of the wheezing episode (day 0: −23.9% ± 12.1% versus baseline: −12% ± 13.5%, *p* < 0.001), irrespectively of atopic status (Table 2 and Figure 2). The same applied for the ΔR(5−20), as depicted in Figure 3. Bronchodilation responses measured with ΔR20 values on day 0 did not differ from the responses recorded at baseline or on days 10 and 30 (Figure 4), therefore excluding any potential diagnostic value of ΔR20 to classify wheezing episodes correctly.

ΔR5 and ΔR(5−20) were additionally examined as potential diagnostic markers of clinically significant increase in peripheral resistance (assessed during a wheezing episode) in comparison with measures during asymptomatic periods (baseline, day 10, and day 30). For this reason, a ROC analysis was performed. The models examined were unadjusted since none of the other parameters was found to be significant.

The AUCs were 0.725 and 0.671 for ΔR5 and ΔR(5–20) (*p* = 0.118). Although the AUCs were not significantly different, the higher number for the ΔR5Hz and the simplicity of its calculation render it preferable.

In the ROC analysis, a value of ΔR5 ≤−46.4% accurately classified 80.6% of the wheezing episodes, while values ≤−35.1% correctly identified 77.3% of them (Supplementary Figure S5). Selected cutoff values for ΔR5 and their respective sensitivity, specificity, positive predictive value (PPV) and negative predictive value (NPV), are presented in Table 3.

## 4. Discussion

To our knowledge, this is the first study to prospectively assess IOS indices in virus-induced wheezing illnesses, suggesting that this method could be used in this age group, where spirometry might not be feasible for a substantial proportion of the children.

The IOS technique is easy to perform in preschoolers. All recruited children were trained within a few minutes, and more importantly, none of them experienced difficulties in cooperating during the episode. On the contrary, during the recruitment period, 44 out of 93 children (47.3%) needed more than a regular (according to the study protocol) visit to be properly trained in order to perform a correct spirometric effort, while two of them did not achieve a technically acceptable spirometric maneuver during the episode. Taking into consideration the fact that measurements were performed in a research setting, it is rather self-explanatory that, in everyday clinical practice, spirometry might be quite time-consuming for the medical personnel, thus indisputably supporting the IOS’s superiority in this age group. Since there is an unmet need for objective measures in preschoolers with asthma-related symptoms, seeing that the documentation of reversibility using spirometry is often problematic due to effort dependency, IOS merits the consideration of being included in the diagnostic and therapeutic algorithm.

In particular, we showed that, in preschool children with virus-induced wheeze, IOS indices mainly reflecting peripheral airways, such as R5, are indicative of airflow limitation during an asthma-associated episode. R5 values increased significantly, while bronchodilation responses were more pronounced upon the initiation of the wheezing illness than at baseline. Fluctuations of the IOS resistance parameters resemble the correspondent fluctuations of symptoms (noisy breathing, cough, and shortness of breath), airway inflammation (FeNO) and spirometric parameters (such as FEV_0.5_)—as shown in our previously published study, indicating that these episodes share common characteristics with the well-defined asthma exacerbations in older children [18].

In general, the within- and between-visit variability of R5 and R20 is estimated to be up to 10%. This variability indicates acceptable repeatability among multiple efforts performed by the same subject [22]. In children, this variability is expected to be higher and has been estimated to be up to 16% for measurements within the same day or up to 30% among several weeks [7,10,19,23,24]. When assessing post-bronchodilation values, their variability is expected to vary even more, with coefficients of variation twice the amount of these reported for measurements before bronchodilation [19]. This was also the case in our cohort. The median variability ranged from 7% to 15% after taking into consideration pre- and post- bronchodilation measurements for R5 and R20 at regular visits and during the course of a wheezing episode. The variability did not differ significantly before and after bronchodilation and, on both occasions, it was within the acceptable extent, suggesting excellent repeatability and reliability in recorded measurements at different time points.

In our cohort, moderate to low correlations (ranging from 0.308 to 0.522) were found between predicted and recorded values for both R5 and R20, before and after bronchodilation. Moreover, neither pre- nor post-bronchodilation R5 and R20 values were found to be correlated with either age, gender, or somatometric parameters. Predicted values for R5 and R20 were based on gender- and height-adjusted existing reference values from a Swedish and a Polish study, as recommended by the manufacturer [20,21]. In the Swedish study by Denker et al. [20], 350 children, 2.1 to 11.1 years old with height 90–160 cm were examined. The investigators found significant correlations with height but weak with weight for both R5 and R20. No significant gender-related difference was found for respiratory resistance. The investigators stated that there were more observations in the height interval 130–145 cm (vs. 107–121 cm in our study population). They do not provide information about the number of children between the 4–6 year of age but based on the height information, it is clear that the predicted equations are weighted for older and higher children and are not representative for preschoolers. In the Polish study by Nowowiejska et al. [21], 626 children, 3.1–18.9 years old (mean age for boys 10.6 years and for girls 10.9 years), with height 95–193 cm (mean height for boys 144.8 cm and for girls 141.9 cm) were examined. Similar correlations with the Swedish study were found for both R5 and R20. Again, the predicted equations were weighted over older and higher children.

In general, young children exhibit higher pulmonary resistance than older children and adults, and, therefore, airway resistance is inversely proportional to age, especially at lower frequencies [25]. In a recent meta-analysis, a correlation with anthropometric variables has been suggested too [26]. The lack of similar correlations in our study could be explained by the narrow range of height and weight in the sample of 4- to 6-year-old children examined. The Polish and Swedish studies provide predicted equations with anthropometric data based mainly on older and higher children, with values over a wide spectrum. Thus, extrapolating data in younger and smaller children appears to provide suboptimal reference values. Additionally, it should be underscored that the differences in ethnicity might also influence the estimated reference values which do not seem to be appropriate at least for the Greek children.

Nevertheless, based on the low variability and excellent repeatability in our study, measures up to six months apart (based on this study average observational period until a wheezing episode occurred), even during different seasons, could be considered stable and independent of known confounders like gender, height or weight. For these reasons, at least in this age-group, reference values are not recommended, until appropriate studies are conducted. An individualized approach using the personal best value, recorded either when the child is asymptomatic or at least 10 days after a mild wheezing episode occurs, is recommended. These values can be used up to at least six months apart to follow-up mild wheezers.

Atopy does not seem to affect the resistance baseline measurements or the bronchodilation responses either when these parameters are assessed cross-sectionally or longitudinally. This finding has also been shown in spirometry and FE_NO_ in preschoolers [18]. This independence could be explained by the intermittent, short-lasting inflammatory responses during mild wheezing episodes in our cases. It remains to be shown [27] whether the probably new, atopy-related inflammation could predispose patients to a more chronic inflammatory process since multiple relapses could lead to alterations in the peripheral resistance measures and bronchodilation responses in atopics.

R5 and R(5−20) values, as indicators of bronchial obstruction, increased significantly during wheezing episodes and spontaneously returned to baseline within 10 days. Post-bronchodilation R5 values on day 0 were similar to the pre-bronchodilation baseline values, reflecting a reversible airway obstruction at the beginning of a wheezing episode, independently of atopic status as has previously been shown for FEV_0.5_ [18].

Mean bronchodilation responses were significantly higher at the beginning of the episode compared to the respective changes at baseline or on day 10 and 30 for ΔR5 (representing the total airway resistance) and ΔR(5−20) (representing peripheral resistance) but not for ΔR20 (representing the resistance of the large airways). Considering the resistance that each of these variables reflects, this is to be expected. In particular, the bronchodilation responses assessed by ΔR5 were more pronounced within the first 48 h from the initiation of the wheezing episode than the responses assessed by ΔR(5−20). When both ΔR5 and ΔR(5−20) were examined for their potential diagnostic value in discriminating clinically significant increase in peripheral resistance during the first days of a wheezing episode (day 0, day 10 and day 30), compared to measurements during asymptomatic periods (baseline) their AUCs were not significantly different (0.725 vs 0.671, respectively). Nevertheless, the higher absolute magnitude of AUC_ΔR5_ renders this parameter preferable for analyzing performance characteristics with a ROC analysis.

Indeed, the ROC analysis provided specific diagnostic cutoff values. Based on the analysis of this cohort, the optimal cutoff point with the highest sensitivity and specificity was found to be −20.5% (Table 3). Taking into account that the cutoff values have been calculated in patients using measurements even during a wheezing episode, the bronchodilation magnitude is expected to be overestimated in healthy individuals of similar age, and, hence, the proposed cutoff values are expected to be of greater diagnostic value.

The major strength of this study is the steroid-naïve cohort that has been longitudinally examined, providing robust data. To our knowledge, this is the first study to assess airflow limitation in preschool children experiencing wheezing episodes with such study design, that is, by utilizing the IOS technique, and it is also the first time cutoff values have been calculated based on longitudinal measures during and outside an episode for a short period, to adjust for intra- and inter-variability. Considering the fact that the two most likely sources to rely on for the calculation of cutoffs are either population surveys—using only non-wheezers—or studies comparing wheezers with non-wheezers during asymptomatic periods, the data of this study are expected to be more reliable.

A weakness of the study is the lack of a healthy control group because of ethical considerations regarding salbutamol administration to healthy children [28]. This issue has been overcome by assessing the value of a personalized approach and proving that the personal best value is reliable enough to follow up with such patients. Moreover, the sample was small, although the multiple measurements per studied child provided robust longitudinal analyses. Last but not least, it is not known if the inferences could be applied in children with severe viral-induced wheezing episodes.

In summary, we have shown that IOS is an easy, highly reproducible, and sensitive technique that can be successfully performed to assess airflow limitation objectively in preschool children with virus-induced wheezing illnesses. The study design supports the superiority of the longitudinal approach of such data, suggesting, that reference or predicted values, although necessary, are suboptimal, and that an individualized approach utilizing the personal best values as the personal reference is useful and reliable. Among the commonly measured parameters, the R5 seems to be the best to assess clinically significant fluctuations and bronchodilation responses suggestive of airflow limitation. Cutoff values do have diagnostic properties that can help identify significant bronchodilation responses. Considering the difficulties in examining preschoolers using spirometry and the time required to train them and perform an acceptable spirometric maneuver, IOS could be suggested as the future gold standard to examine airflow limitation in children even in everyday clinical practice. Further studies with similar design are needed for children with persistent or moderate to severe symptoms.

## Figures and Tables

**Table 1:** Baseline characteristics of children experiencing a wheezing episode during the study period, and children without a wheezing episode during the study period.

	Children with Wheezing Episode * (*n* = 43)	Children with no Episode † (*n* = 46)
**Age (years)**	5 ± 0.5	5 ± 0.7
**Male, n (%)**	23 (54%)	20 (44%)
**Height (m)**	1.15 ± 0.08	1.14 ± 0.08
**Weight (kg)**	22.9 ± 3.9	21.9 ± 4.3
**Atopic**	25 (58%)	21 (46%)
**Baseline pre-bronchodilation R5Hz** (kPa/lt/sec)	0.943 ± 0.269	0.980 ± 0.222
**Baseline post-bronchodilation R5Hz** (kPa/lt/sec)	0.817 ± 0.227	0.809 ± 0.186
**ΔR5Hz**	−12% ± 13.5%	−15.8% ± 15.4%
**Baseline pre-bronchodilation R20Hz** (kPa/lt/sec)	0.757 ± 0.191	0.764 ± 0.173
**Baseline post-bronchodilation R20Hz** (kPa/lt/sec)	0.669 ± 0.157	0.675 ± 0.153
**ΔR20Hz**	−10.1% ± 13.9%	−11.5% ± 17.2%
**Previously treated with** **Bronchodilators alone ^§^**	32 (74.4%)	32 (69.6%)

Values presented as mean ± (standard deviation) SD. All comparisons are non-significantly different. * baseline values obtained eight weeks prior to the recorded wheezing episode; † baseline values obtained at recruitment and atopic status at the age of six years old; ^§^ the rest of the children had been treated prior to enrolment with inhaled corticosteroids and/or montelukast; Pulmonary resistance (R) at 5 Hz (R5) and 20 Hz (R20); ΔRx = (Rx_post-bronchodilation_ − Rx_pre-bronchodilation_)/Rx_pre-bronchodilation._

**Table 2:** Mean R5Hz pre- and post-bronchodilation in atopic and non-atopic children and percentage of response to bronchodilation: baseline and day 0, 10, and 30 from the beginning of the wheezing episode.

	Atopics			Non-Atopics		
	R5Hz Bronchodilation (kPa/lt/sec)		Mean ΔR5Hz%*	R5Hz Bronchodilation (kPa/lt/sec)		Mean ΔR5Hz%*
Time	Pre	Post		Pre	Post	
**Baseline**	0.930 ± 0.273	0.798 ± 0.230	−12.8% ± 14.9%	0.961 ± 0.271	0.844 ± 0.227	−10.8% ± 11.6%
**Day 0**	1.106 ± 0.279	0.847 ± 0.218 †	−22.5 ± 12.8% ‡	1.125 ± 0.289	0.818 ± 0.171 †	−25.8% ± 11.1% ‡
**Day 10**	0.959 ± 0.225 †	0.831 ± 0.218 †	−12.9% ± 13.5%	0.914 ± 0.192	0.803 ± 0.155 †	−11.2% ± 11.3%
**Day 30**	0.905 ± 0.248 †	0.792 ± 0.159 †	18.6% ± 10.7%	0.947 ± 0.179	0.772 ± 0.191 †	−10.3% ± 12.6%

All bronchodilation responses are significantly different at all time points. ΔR5 = (R5_post-bronchodilation_ − R5_pre-bronchodilation_) / R5_pre-bronchodilation_; † non-significant results compared with the respective (atopics, non-atopics) baseline pre-bronchodilation values; ‡ Significant bronchodilation differences when compared with the respective (atopics, non-atopics) baseline ΔR5Hz (*p*-value= 0.008 and *p*-value <0.001 respectively). All other comparisons with baseline ΔR5Hz are not significant, irrespectively of atopic status.

**Table 3:** Positive and negative predicted values, sensitivity and specificity between selected ΔR5Hz values, and their ability to correctly classify wheezing episodes (or increase in peripheral resistance as it is assessed during a wheezing episode).

ΔR5Hz (%)	PPV	NPV	Sensitivity	Specificity	Accuracy
−46.4%	100.00%	80.38%	4.65%	100.00%	80.57%
−35.1%	30.77%	80.30%	9.30%	94.64%	77.25%
−31.3%	32.00%	81.18%	18.60%	89.88%	75.36%
−20.5%	42.25%	90.71%	69.77%	75.60%	74.41%
−12.4%	28.57%	90.22%	79.07%	49.40%	55.45%
−8.3%	26.03%	92.31%	88.37%	35.71%	46.45%
−7.1%	25.16%	94.23%	93.02%	29.17%	42.18%

Data are given as percentages. PPV = Positive predictive value of having a wheezing episode if ΔR5Hz is lower than or equal to that specified in the first column; NPV = negative predictive value of not having a wheezing episode if ΔR5Hz is higher than or equal to that specified in the first column.

## Supplementary Materials

The following are available online at https://www.mdpi.com/2077-0383/8/9/1475/s1, Figure S1: Pairwise correlation between reference/predicted values and actual values for pre-bronchodilation R5, Figure S2: Pairwise correlation between reference/predicted values and actual values for pre-bronchodilation R20, Pairwise correlation between reference/predicted values and actual values for post-bronchodilation R5, Figure S4: Pairwise correlation between reference/predicted values and actual values for post-bronchodilation R20, Figure S5: Receiver operating characteristic (ROC) curve, Table S1. Baseline characteristics of atopic and non-atopic children with a wheezing episode and children without a wheezing episode during the study period, Table S2. Pre-bronchodilation R5Hz measurements: reference (baseline), on day 0, 10th (day 10), and 30th (day 30) from the beginning of the wheezing episode.

## References

[1] Global Initiative for Asthma (2019). Global Strategy for Asthma Management and Prevention. www.ginasthma.org.

[2] Bickel S., Popler J., Lesnick B., Eid N. (2014). Impulse oscillometry: Interpretation and practical applications. Chest.

[3] Meraz E.G., Nazeran H., Ramos C.D., Nava P., Diong B., Goldman M.D., Goldman C.A. (2011). Analysis of impulse oscillometric measures of lung function and respiratory system model parameters in small airway-impaired and healthy children over a 2-year period. Biomed. Eng. Online.

[4] Song T.W., Kim K.W., Kim E.S., Park J.W., Sohn M.H., Kim K.E. (2008). Utility of impulse oscillometry in young children with asthma. Pediatric Allergy Immunol. Off. Publ. Eur. Soc. Pediatric Allergy Immunol..

[5] Shi Y., Aledia A.S., Tatavoosian A.V., Vijayalakshmi S., Galant S.P., George S.C. (2012). Relating small airways to asthma control by using impulse oscillometry in children. J. Allergy Clin. Immunol..

[6] Shi Y., Aledia A.S., Galant S.P., George S.C. (2013). Peripheral airway impairment measured by oscillometry predicts loss of asthma control in children. J. Allergy Clin. Immunol..

[7] Komarow H.D., Skinner J., Young M., Gaskins D., Nelson C., Gergen P.J., Metcalfe D.D. (2012). A study of the use of impulse oscillometry in the evaluation of children with asthma: analysis of lung parameters, order effect, and utility compared with spirometry. Pediatric Pulmonol..

[8] Dos Santos K., Fausto L.L., Camargos P.A.M., Kviecinski M.R., da Silva J. (2017). Impulse oscillometry in the assessment of asthmatic children and adolescents: From a narrative to a systematic review. Paediatr. Respir. Rev..

[9] Frei J., Jutla J., Kramer G., Hatzakis G.E., Ducharme F.M., Davis G.M. (2005). Impulse oscillometry: Reference values in children 100 to 150 cm in height and 3 to 10 years of age. Chest.

[10] Klug B., Bisgaard H. (1998). Specific airway resistance, interrupter resistance, and respiratory impedance in healthy children aged 2–7 years. Pediatric Pulmonol..

[11] Hellinckx J., De Boeck K., Bande-Knops J., van der Poel M., Demedts M. (1998). Bronchodilator response in 3–6.5 years old healthy and stable asthmatic children. Eur. Respir. J..

[12] Thamrin C., Gangell C.L., Udomittipong K., Kusel M.M., Patterson H., Fukushima T., Schultz A., Hall G.L., Stick S.M., Sly P.D. (2007). Assessment of bronchodilator responsiveness in preschool children using forced oscillations. Thorax.

[13] Mansur A.H., Manney S., Ayres J.G. (2008). Methacholine-induced asthma symptoms correlate with impulse oscillometry but not spirometry. Respir. Med..

[14] Kalliola S., Malmberg L.P., Kajosaari M., Mattila P.S., Pelkonen A.S., Mäkelä M.J. (2014). Assessing direct and indirect airway hyperresponsiveness in children using impulse oscillometry. Ann. Allergy Asthma Immunol. Off. Publ. Am. Coll. Allergy Asthma Immunol..

[15] Brand P.L., Baraldi E., Bisgaard H., Boner A.L., Castro-Rodriguez J.A., Custovic A., de Blic J., de Jongste J.C., Eber E., Everard M.L. (2008). Definition, assessment and treatment of wheezing disorders in preschool children: An evidence-based approach. Eur. Respir. J..

[16] Bacharier L.B., Boner A., Carlsen K.H., Eigenmann P.A., Frischer T., Götz M., Helms P.J., Hunt J., Liu A., Papadopoulos N. (2008). Diagnosis and treatment of asthma in childhood: A PRACTALL consensus report. Allergy.

[17] Beydon N., Davis S.D., Lombardi E., Allen J.L., Arets H.G., Aurora P., Bisgaard H., Davis G.M., Ducharme F.M., Eigen H. (2007). An official American Thoracic Society/European Respiratory Society statement: Pulmonary function testing in preschool children. Am. J. Respir. Crit. Care Med..

[18] Konstantinou G.N., Xepapadaki P., Manousakis E., Makrinioti H., Kouloufakou-Gratsia K., Saxoni-Papageorgiou P., Papadopoulos N.G. (2013). Assessment of airflow limitation, airway inflammation, and symptoms during virus-induced wheezing episodes in 4- to 6-year-old children. J. Allergy Clin. Immunol..

[19] Oostveen E., MacLeod D., Lorino H., Farré R., Hantos Z., Desager K., Marchal F. (2003). The forced oscillation technique in clinical practice: Methodology, recommendations and future developments. Eur. Respir. J..

[20] Dencker M., Malmberg L.P., Valind S., Thorsson O., Karlsson M.K., Pelkonen A., Pohjanpalo A., Haahtela T., Turpeinen M., Wollmer P. (2006). Reference values for respiratory system impedance by using impulse oscillometry in children aged 2–11 years. Clin. Physiol. Funct. Imaging.

[21] Nowowiejska B., Tomalak W., Radliński J., Siergiejko G., Latawiec W., Kaczmarski M. (2008). Transient reference values for impulse oscillometry for children aged 3–18 years. Pediatric Pulmonol..

[22] Lándsér F.J., Nagles J., Demedts M., Billiet L., van de Woestijne K.P. (1976). A new method to determine frequency characteristics of the respiratory system. J. Appl. Physiol..

[23] Gochicoa-Rangel L., Torre-Bouscoulet L., Martínez-Briseño D., Rodríguez-Moreno L., Cantú-González G., Vargas M.H. (2015). Values of impulse oscillometry in healthy Mexican children and adolescents. Respir. Care.

[24] Knihtilä H., Kotaniemi-Syrjänen A., Pelkonen A.S., Kalliola S., Mäkelä M.J., Malmberg L.P. (2017). Small airway oscillometry indices: Repeatability and bronchodilator responsiveness in young children. Pediatric Pulmonol..

[25] Clément J., Dumoulin B., Gubbelmans R., Hendriks S., van de Woestijne K.P. (1987). Reference values of total respiratory resistance and reactance between 4 and 26 Hz in children and adolescents aged 4–20 years. Bull. Eur. De Physiopathol. Respir..

[26] De Assumpcao M.S., da Silva Goncalves E., Oliveira M.S., Ribeiro J.D., Dalbo Contrera Toro A.A., de Azevedo Barros-Filho A., de Monteiro M., Santos Schivinski C.I. (2017). Impulse Oscillometry System and Anthropometric Variables of Preschoolers, Children and Adolescents Systematic Review. Curr. Pediatric Rev..

[27] Xepapadaki P., Bachert C., Finotto S., Jartti T., Konstantinou G.N., Kiefer A., Kowalski M., Lewandowska-Polak A., Lukkarinen H., Roumpedaki E. (2018). Contribution of repeated infections in asthma persistence from preschool to school age: Design and characteristics of the PreDicta cohort. Pediatric Allergy Immunol. Off. Publ. Eur. Soc. Pediatric Allergy Immunol..

[28] Lands L.C., Allen J., Cloutier M., Leigh M., McColley S., Murphy T., Wilfond B. (2005). Pediatric Assembly of American Thoracic Society Subcommittee. ATS Consensus Statement: Research opportunities and challenges in pediatric pulmonology. Am. J. Respir. Crit. Care Med..

