# Supplementary material for: Virus-Induced Asthma/Wheeze in Preschool Children: Longitudinal Assessment of Airflow Limitation Using Impulse Oscillometry
Source: J Clin Med. 2019 Sep 16;8(9):1475. doi: 10.3390/jcm8091475 (PMC6780792; doi:10.3390/jcm8091475)
Supplement: Supplementary file 1 [file jcm-08-01475-s001.pdf]

Methods

Statistical Analysis

R5, R20, and R(5−20) are highly correlated (correlation coefficients 0.797–0.9315) to preclude coexistence in the same model due to co-linearity introduction.

Analyses across these time points were performed with generalized estimating equations (GEE) after adjusting for known confounders, such as height, age, and atopy, by using the Gaussian family of distribution and an unstructured correlation [1].

Results

There is a rule of thumb for interpreting the size of a correlation coefficient in which values of 0.9–1 are characterized as very high, 0.7–0.9 as high, 0.5–0.7 as moderate, 0.3–0.5 as low, and 0–0.3 as negligible correlations [1]. The correlation coefficients calculated for the reference values versus the actual values measured before or after bronchodilation for the R5 and R20 were 0.522, 0.496, 0.415, and 0.308, respectively (Supplementary Figures S1–S4). These low correlations suggest suboptimal reference values which were not used either to produce Z-scores or in any other calculation.

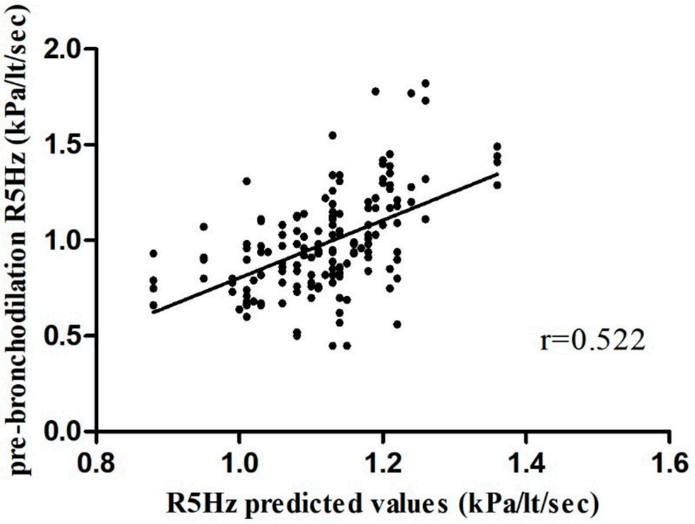

Supplementary Figure S1. Pairwise correlation between reference/predicted values and actual values for pre-bronchodilation R5.

r: Pearson's correlation coefficient

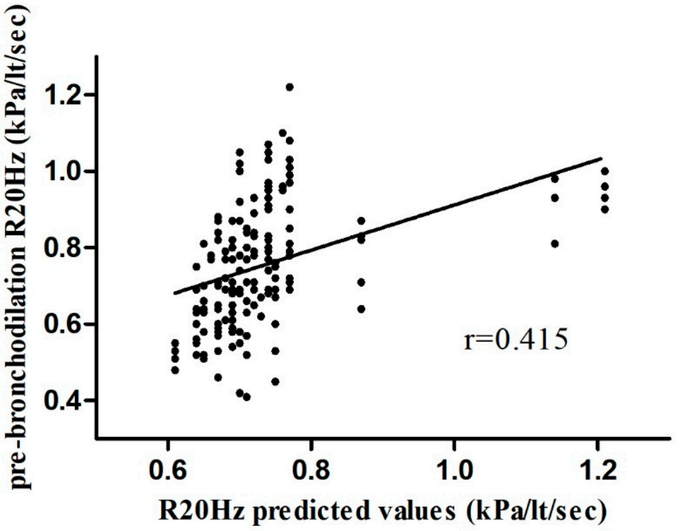

Supplementary Figure S2. Pairwise correlation between reference/predicted values and actual values for pre-bronchodilation R20.

r: Pearson's correlation coefficient

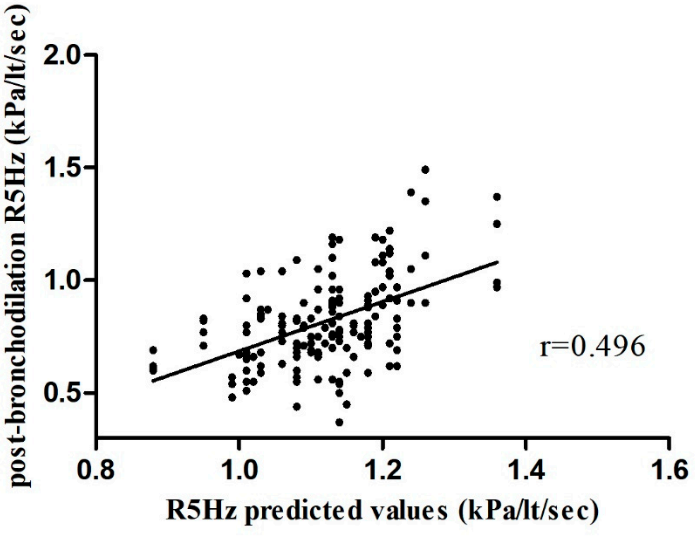

Supplementary Figure S3. Pairwise correlation between reference/predicted values and actual values for post-bronchodilation R5.

r: Pearson's correlation coefficient

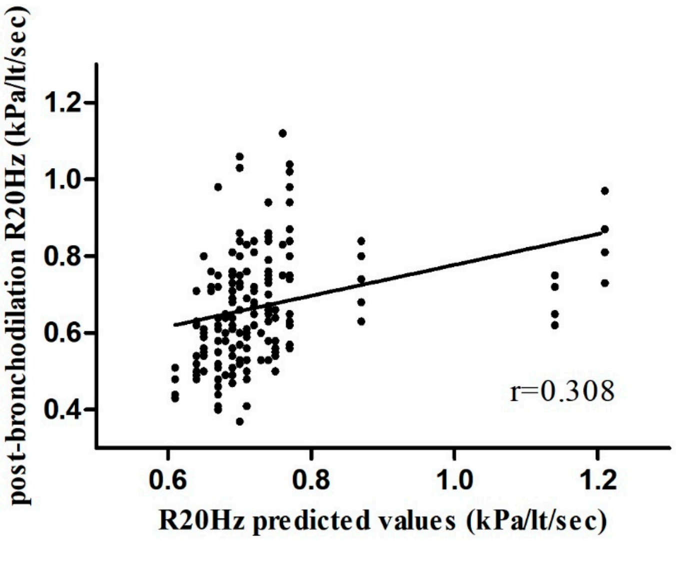

Supplementary Figure S4. Pairwise correlation between reference/predicted values and actual values for post-bronchodilation R20.

r: Pearson's correlation coefficient

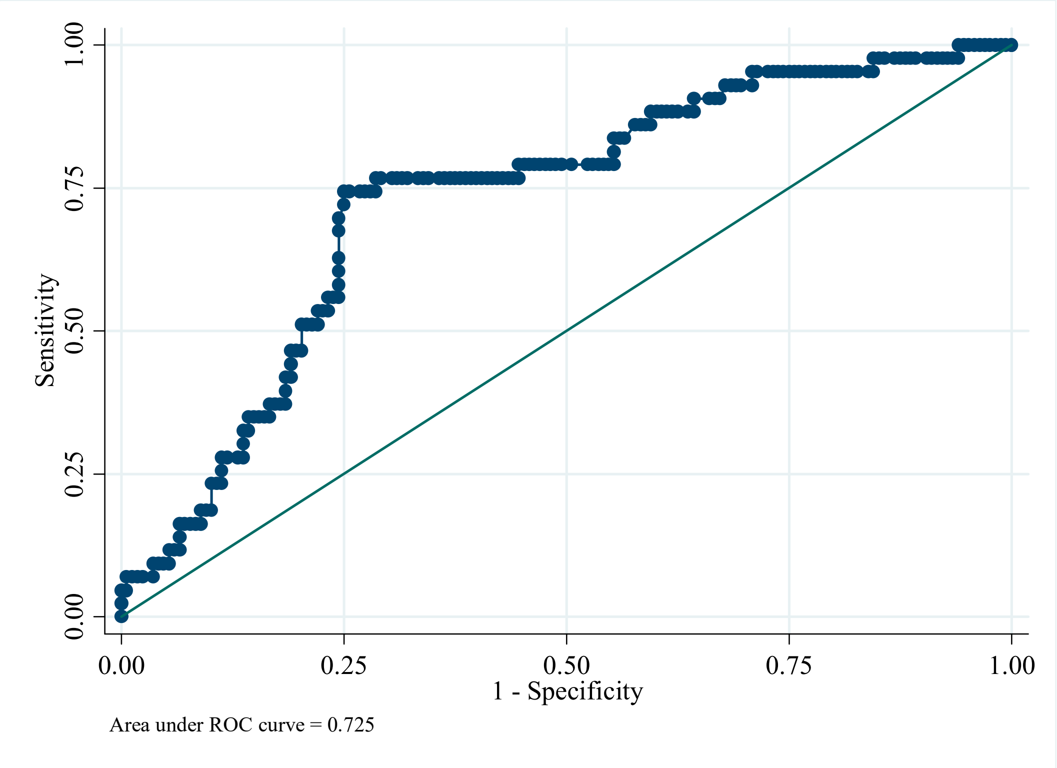

Supplementary Figure S5. Receiver operating characteristic (ROC) curve.

Supplementary Table S1. Baseline characteristics of atopic and non-atopic children with a wheezing episode and children without a wheezing episode during the study period.

	**Atopic Wheezers * (*n* = 25)**	**Non-Atopic Wheezers * (*n* = 18)**	**Children with no Episode † (*n* = 46)**
**Age (years)**	5 ± 0.5	5 ± 0.6	5 ±0.7
**Male, n (%)**	14 (56%)	9 (50%)	20 (44%)
**Height (m)**	1.11 ± 0.07	1.15 ± 0.05	1.14 ± 0.08
**Weight (kg)**	24.91 ± 3.98	21.44 ±3 .01	21.87 ± 4.28
**Atopic**	25	0	21 (46%)
**Baseline pre-bronchodilation R5Hz** (kPa/lt/sec)	0.930 ± 0.273	0.961 ±0.271	0.980 ± 0.222
**Baseline post-bronchodilation R5Hz** (kPa/lt/sec)	0.798 ± 0.230	0.844 ± 0.227	0.809 ± 0.186
**ΔR5Hz**	−12.8% ± 14.9%	−10.8% ± 11.6%	−15.8% ±15.4%
**Baseline pre-bronchodilation R20Hz** (kPa/lt/sec)	0.757 ± 0.189	0.757 ± 0.199	0.764 ± 0.173
**Baseline post-bronchodilation R20Hz** (kPa/lt/sec)	0.663 ± 0.148	0.677 ± 0.172	0.675 ± 0.153
**ΔR20Hz**	−10.8% ± 11.7%	−9.2% ± 13%	−11.5% ± 17.2%
**Previously treated with Bronchodilators alone§**	18 (72%)	14 (77.8%)	32 (69.6%)

Values presented as mean ± standard deviation (SD). All comparisons are non-significantly different. * baseline values obtained eight weeks prior to the recorded wheezing episode; † baseline values obtained at recruitment and atopic status at age of six years old; § the rest of the children had been treated prior to enrolment with inhaled corticosteroids and/or montelukast; $\Delta Rx = (Rx_{post\text{-}bronchodilation} - Rx_{pre\text{-}bronchodilation}) / Rx_{pre\text{-}bronchodilation}$.

Supplementary Table S2. Pre-bronchodilation R5Hz measurements: reference (baseline), on day 0, 10th (day 10), and 30th (day 30) from the beginning of the wheezing episode.

R5Hz values	Atopics	Non-Atopics	*p*-value †
**Baseline** (kPa/lt/sec)	0.930 ± 0.273	0.961 ± 0.271	0.740
**Day 0** (kPa/lt/sec)	1.106 ± 0.279	1.125 ± 0.289	0.758
p*-value (Baseline vs Day 0)	0.002	0.010	
**Day 10** (kPa/lt/sec)	0.959 ± 0.225	0.914 ± 0.192	0.588
p*-value (Baseline vs Day 10)	0.667	0.316	
p*-value (Day 0 vs Day 10)	<0.001	<0.001	
**Day 30** (kPa/lt/sec)	0.905 ± 0.248	0.947 ± 0.179	0.431
p*-value (Baseline vs Day 30)	0.710	0.605	
p*-value (Day 0 vs Day 30)	<0.001	0.002	
p*-value (Day 10 vs Day 30)	0.317	0.301	

Values are presented as mean ± SD. **p*-values have been estimated with paired Student's t-test.
† *p*-values between atopics and non-atopics have been estimated with Student's t-test.

e-References

1. Zeger, S.L.; Liang, K.Y. Longitudinal data analysis for discrete and continuous outcomes. *Biometrics* **1986**, *42*, 121-130.

2 Hinkle, D.E.; Wiersma, W.; Jurs, S.G. *Applied Statistics for the Behavioral Sciences*. 5th ed. Boston: Houghton Mifflin **2003**.
